# Decoding Images in the Mind’s Eye: The Temporal Dynamics of Visual Imagery

**DOI:** 10.3390/vision3040053

**Published:** 2019-10-21

**Authors:** Sophia M. Shatek, Tijl Grootswagers, Amanda K. Robinson, Thomas A. Carlson

**Affiliations:** 1 School of Psychology, University of Sydney, Sydney, NSW 2006, Australia; tijl.grootswagers@sydney.edu.au (T.G.); amanda.robinson@sydney.edu.au (A.K.R.); thomas.carlson@sydney.edu.au (T.A.C.); 2 ARC Centre of Excellence in Cognition & Its Disorders, Macquarie University, Sydney, NSW 2109, Australia; 3 Perception in Action Research Centre & Department of Cognitive Science, Macquarie University, Sydney, NSW 2109, Australia

**Keywords:** mental imagery, electroencephalography, MVPA, decoding

## Abstract

Mental imagery is the ability to generate images in the mind in the absence of sensory input. Both perceptual visual processing and internally generated imagery engage large, overlapping networks of brain regions. However, it is unclear whether they are characterized by similar temporal dynamics. Recent magnetoencephalography work has shown that object category information was decodable from brain activity during mental imagery, but the timing was delayed relative to perception. The current study builds on these findings, using electroencephalography to investigate the dynamics of mental imagery. Sixteen participants viewed two images of the Sydney Harbour Bridge and two images of Santa Claus. On each trial, they viewed a sequence of the four images and were asked to imagine one of them, which was cued retroactively by its temporal location in the sequence. Time-resolved multivariate pattern analysis was used to decode the viewed and imagined stimuli. Although category and exemplar information was decodable for viewed stimuli, there were no informative patterns of activity during mental imagery. The current findings suggest stimulus complexity, task design and individual differences may influence the ability to successfully decode imagined images. We discuss the implications of these results in the context of prior findings of mental imagery.

## 1. Introduction

Does the Mona Lisa face left or right? A common method of solving this problem is to form an image of the Da Vinci painting in your ‘mind’s eye’. Our ability to imagine scenes and objects can help us solve everyday problems and accomplish day-to-day tasks, such as retracing our steps to find a lost item or navigating from a memorised map. These mentally-generated images are formed in the absence of visual information and are instead based on short- or long-term memories [1,2]. Images generated from memory seem anecdotally weaker, or less vivid, than those evoked by sensory input, yet also appear to rely on the visual system [3]. In line with this, current theories of mental imagery involve common mechanisms for human vision and mental imagery.

Recent work has revealed overlapping neural substrates for visual perception and imagery. Positron emission tomography (PET) and functional magnetic resonance imaging (fMRI) have revealed similar patterns of brain activity during perception and imagery, suggesting computational overlap in the neural systems responsible for each process [4,5,6,7]. This overlap is particularly clear for areas associated with higher-order abstract visual processing, such as visual association cortex [8,9,10] and category-selective temporal cortices [11,12,13]. Overlapping activation is also present in low-level visual areas, despite the absence of visual input during imagery; imagery and visual perception both activate the lateral geniculate nucleus of the thalamus (LGN) [14] and primary visual cortex (V1) [8,15,16]. Together, this supports the notion that imagery utilises many of the same mechanisms as visual perception.

Despite overlapping neural activation for vision and imagery, the neural processes are not identical. For example, there is more overlap in higher, anterior regions (i.e., frontal and parietal [4]), compared to lower, posterior visual regions [6,15]. There are also task-related differences in imagery such that different imagery tasks show varying degrees of overlap with vision [4,17,18]. Patients with brain damage also provide evidence for dissociation between imagery and vision. Some patients with occipital or parietal lesions can successfully complete tasks relying on mental imagery, despite significant visual deficits, while others have fully functioning vision but impaired imagery [19,20,21,22]. Therefore, there is some dissociation between vision and imagery despite similar neural processing.

To date, research has focused on understanding the brain networks recruited by a variety of imagery tasks [11,23], yet we have very little understanding of the temporal dynamics of mental imagery. Although fMRI studies have found correlations between imagery and perception in the later stages of visual processing [24], as well as similar activation patterns between imagery and working memory [8], this evidence is limited by the temporal resolution of fMRI. Recent work using MEG has revealed that while similar activation patterns are present in imagery and vision, predictable activation patterns during imagery occur at a later time and are more diffuse than vision. This hints towards some degree of temporal dissociation between the two seemingly similar processes [3].

Multi-Variate Pattern Analysis (MVPA) applied to neuroimaging data can elucidate the information represented in different brain regions (fMRI) and at particular points in time (M/EEG). MVPA offers an advantage in analysing data from mental imagery, as analyses are conducted at an individual-subject level, and mental imagery ability is understood to vary significantly between people (e.g., [25]). MVPA is also more sensitive to variation across fine-grained patterns and provides a powerful framework for the detection of content-specific information [26,27]. This is particularly advantageous for imagery signals that are likely to be weaker than visual input [28]. One recent study found that the category of imagined images (faces and houses) was decodable from MEG recordings, albeit later than viewed images [3]. However, decoding of individual exemplars was poor, indicating a dissociation between low- and high-level imagery processes.

Here, we examined how the neural representation of mental images develops and changes over time. Participants imagined one of four previously learned pictures: two faces and two places. Each image was visually dissimilar to the other within the category, while maintaining clear category divisions. Neural responses were measured using EEG while participants viewed the experimental images, imagined the images, and viewed fast streams of semantically related images (i.e., other faces and places). We expected that category information would be decodable from the EEG data during mental imagery, that it would be broadly generalisable across the imagery period, and delayed relative to vision [3]. We also predicted that exemplars within each category would be distinguishable (i.e., successful within-category decoding), based on prior studies of object categorization [29] and the known similarities between vision and mental imagery (e.g., [6]). We found that the dynamics of imagery processes are more variable across and within participants compared to perception of physical stimuli. Although category and exemplar information were decodable for viewed stimuli, there were no informative patterns of activity during mental imagery.

## 2. Materials and Methods

### 2.1. Experimental Structure

At the start of the session, participants completed the Vividness of Visual Imagery Questionnaire (VVIQ) [30]. They were then informed of the task instructions and completed 24 imagery task training trials. The experiment itself consisted of four blocks that were completed while EEG was measured. In each block, participants passively viewed five rapid streams of images (Pattern Estimator), followed by a series of imagery trials. Each imagery trial consisted of a four-image sequence (seen images), after which participants were cued to imagine one of those stimuli (Imagery).

### 2.2. Participants

We recruited 16 right-handed subjects (11 male), of mean age 23 (SD = 5.58, range 18–39), with normal or corrected-to-normal vision and no history of psychiatric or neurological disorders. The experiment was approved by the Human Ethics Committee of the University of Sydney (approval code 2016/849). Written, informed consent was obtained from all participants.

### 2.3. Behavioural Data

To measure individual variation in vividness, we administered a modified VVIQ [30] prior to EEG set-up. The VVIQ measures subjective perception of the strength of an individual’s mental imagery. Participants were asked to imagine 16 scenarios and rate each for vividness on a five-point Likert-like scale. A reversed scoring system was used to decrease confusion. Participants rated each item from 1 (“*No image at all, you only ‘know’ that you are thinking of an object*”) to 5 (“*Perfectly clear and as vivid as normal vision”*). All questions were completed twice, once with open eyes and once with closed eyes. A final summed score between 32 and 160 was calculated for each subject; higher scores indicate greater vividness.

### 2.4. Apparatus and Stimuli

**Figure 1:**
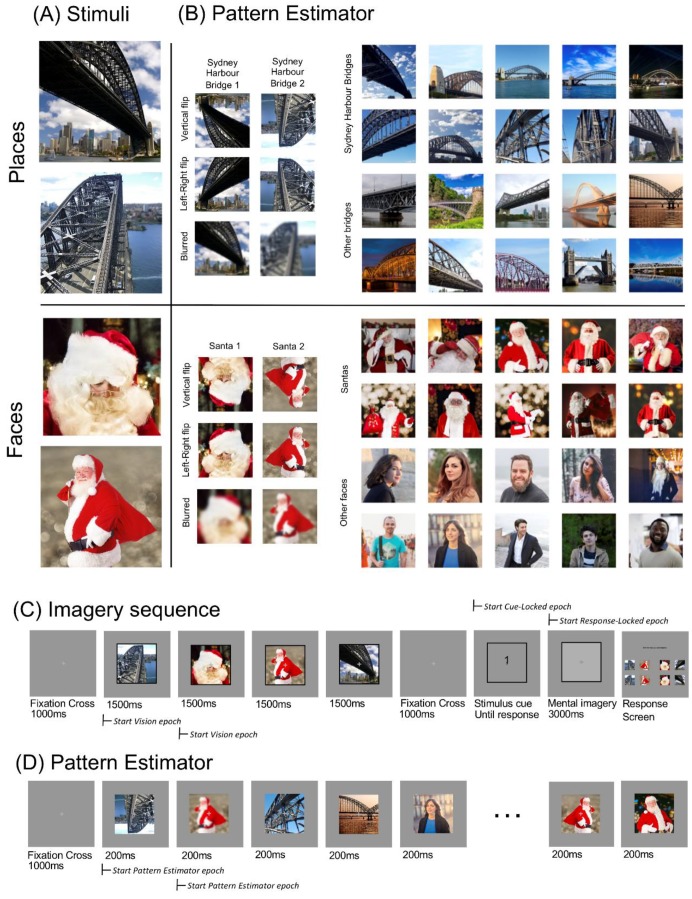
Experimental stimuli and structure. (**A**) The four imagery stimuli. These four images were displayed in every imagery sequence, in a random order, for 1500 ms each. (**B**) Stimuli shown in fast sequences as part of the Pattern Estimator. (**C**) Imagery sequence. Participants viewed a fixation cross for 1000 ms, followed by the four stimuli, each displayed for 1500 ms in a random order. After another 1000 ms fixation cross, a cue (1–4) appeared on the screen, indicating the target to be imagined. Participants clicked to advance when they were projecting a mental image into the frame. After 3000 ms, participants were shown a response screen containing all four stimuli and flipped versions of these stimuli and were asked to click the image they imagined. (**D**) Pattern Estimator. Participants viewed a fixation cross for 1000 ms, followed by a sequence of 56 images, each displayed for 200 ms. Images were sourced online from Creative Commons Zero License sites www.pixabay.com, www.pngimg.com and www.pexels.com.

Four stimuli were used in this experiment: two images of Santa and two images of the Sydney Harbour Bridge (Figure 1A). The inclusion of two exemplars per category allowed us to disentangle whether participants are thinking of the concept (i.e., Santa, Sydney Harbour Bridge) or generating a specific image. These stimuli also fit into distinct face/place categories, which have been shown to evoke robustly distinct patterns of neural activity [31,32].

All stimuli were displayed on a 1920 × 1080-pixel Asus monitor on a grey background. Participants viewed stimuli at approximately 57 cm, such that all stimuli subtended approximately 4.1 degrees of visual angle (including a 0.15-degree black border). Responses were made using a mouse with the right hand. A grey fixation cross was superimposed on all stimuli, with horizontal and vertical arms subtending approximately 0.6 degrees of visual angle. Experimental presentations were coded in MATLAB using extensions from the PsychoPhysics Toolbox [33,34,35].

### 2.5. Imagery Sequence

The experimental structure is shown in Figure 1C. Each imagery sequence began with a fixation cross in the centre of the screen for 1000 milliseconds. The four stimuli (Figure 1A) were displayed sequentially in the centre of the screen, within a black border. Each was displayed for 1500 milliseconds each, in a pseudo-random order. Targets were counterbalanced such that each block contained all 24 possible sequences of the four stimuli. For each sequence, a different target was selected in each block. Target allocation in each block was also randomised. This counterbalancing meant each image appeared in each temporal position as a target equally often.

The fourth stimulus was followed by a 1000 ms fixation cross, then a numerical cue appeared (1–4). This cue referred to the target’s position in the stream; for example, ‘3′ indicated the target was the third image in the stream. Participants were instructed to click the mouse once they had identified the target and were mentally “projecting an image into the square”. Upon clicking, the number was replaced with a dark grey fixation cross, and the frame was filled light grey. This ‘imagery’ screen was displayed for 3000 ms before automatically advancing to a response screen. On the response screen, participants were shown the four stimuli and horizontal mirror images of these stimuli. They used a mouse to select which of these images they were imagining. Mirror images were used as distractors because they are semantically identical but visually different, to determine if participants were using a semantic strategy rather than an imagery-based strategy. Horizontal positioning changed across blocks (stimulus identity), and vertical positioning was randomised every trial (mirror images/stimulus) such that for some trials the mirror image was in the top row, and some in the bottom row. This randomisation aimed to reduce predictability in responses.

### 2.6. Training

Participants completed a block of 24 practice trials of the imagery sequence before EEG recording. We expected these training trials to give participants the opportunity to learn task structure and observe more details about the images to facilitate vivid imagery. Training trials were similar to experimental trials (Figure 1C). The first 12 trials contained typed instructions on how to identify the target and went straight to the response screen after the cue, with no imagery component. On incorrect responses, participants were shown the correct image. The second 12 trials mimicked experimental trials, with the addition of typed instructions and feedback. Instructions displayed on the screen indicated that participants should focus on projecting an image into the designated square. Participants were verbally instructed to visualize the image as vividly as possible, with as much detail as they could. Participants were given the option to repeat the training, and two did so.

### 2.7. Pattern Estimator

We also included a Pattern Estimator at the beginning of each block to investigate the degree of generalisation across semantic category. Images presented during the pattern estimator were semantically similar to the critical experimental stimuli. Participants passively viewed a rapid stream containing the four stimuli from the imagery sequence, as well as horizontally flipped, inverted and blurred versions of these images (Figure 1B, left). It also included other images of the Sydney Harbour Bridge and Santa, other bridges and other people (Figure 1B, right). Each block began with five short streams of 56 images, displayed for 200 ms each (Figure 1D). Every stream contained all 56 images (Figure 1B) in a random order, and lasted for 11.2 s. Participants could pause between streams and elected to advance when they were ready.

### 2.8. Data Recording and Processing

#### 2.8.1. EEG Recording

EEG data were continuously recorded at 1000 Hz using a 64-channel Brain Products (GmbH, Herrsching, Germany) ActiCAP system with active electrodes. Electrode locations corresponded to the modified 10–10 international system for electrode placement [36], with the online reference at Cz. Electrolyte gel kept impedances below 10 kΩ.

#### 2.8.2. Pre-Processing EEG

EEG pre-processing was completed offline using EEGLAB [37] and ERPLAB [38]. The data were minimally pre-processed. Data were down-sampled to 250 Hz to reduce computational load, then filtered using a 0.1 Hz high-pass filter, and a 100 Hz low-pass filter. Line noise at 50 Hz was removed using the CleanLine function in EEGLAB. Four types of epochs were created: Pattern Estimator, Vision, Cue-Locked Imagined and Response-Locked Imagined. Each epoch included 300 ms before to 1500 ms after stimulus onset. Pattern Estimator epochs were from the fast stream at the beginning of each block, from 300 ms before each stimulus appeared, until 1500 ms after the onset. As images were only displayed for 200 ms, these epochs also included the display of the subsequent seven images in the stream. The Vision epochs were taken from the four images displayed in each experimental trial and captured only one of the target stimuli per epoch. Cue-locked Imagined epochs were centred around presentation of the numerical cue designating the target (shown immediately after the four target images, see Figure 1C), beginning 300 ms before the cue appeared on the screen and lasting for 1500 ms after this point. Response-Locked Imagined epochs were centred around participants’ mouse click to begin imagery, beginning 300 ms before the mouse-click and continuing 1500 ms into the imagery period. If the response to the cue was less than 1500 ms, the Cue-Locked and Response-Locked imagery periods would overlap. Although the period between cue and response was variable across trials (Supplementary Figure S1), we expected the period immediately following the cue to provide insight into the initial stages of imagery generation.

#### 2.8.3. Decoding Analysis

All EEG analyses were performed using time-resolved decoding methods, custom-written using CoSMoMVPA functions in MATLAB [39]. For all decoding analyses, a regularised linear discriminant classifier (as implemented in CoSMoMVPA) was trained to differentiate brain patterns evoked by each image or category of images.

For category decoding, a classifier was trained to distinguish images of Santa from images of the Sydney Harbour Bridge for recordings from the same type (i.e., a classifier trained on data from the Pattern Estimator was tested on another independent portion of the Pattern Estimator data). To determine if exemplars were also uniquely represented, a classifier was trained to distinguish between the two exemplars within each category (e.g., decode the two Santa images). Classifiers were trained and tested for each time point using a 12 ms sliding time window (three time points).

To analyse data from the Pattern Estimator and Vision epochs, each presentation sequence was treated as independent. We used a leave-one-trial-out cross-validation approach, where Vision trials were composed of the four stimuli in each imagery sequence and Pattern Estimator trials were composed of a single sequence containing all 56 semantically relevant images. Imagined stimuli were analysed using a leave-two-out cross-validation approach, which took each imagery epoch as independent and left one exemplar of each category (one Santa and one Sydney Harbour Bridge) in the test set. Cross-decoding analyses were conducted using a leave-one-out cross-validation for imagery and vision cross-overs to avoid auto-correlations in the signal if the epochs from the same trial are used to train and test the classifier. All other cross-decoding used a split-half method, where a classifier was trained on one trial type and tested on another trial type (e.g., train on all Vision trials and test on all Cue-Locked Imagined trials). To investigate the possibility of similar processes occurring in vision and imagery at different times, we used temporal generalisation methods [40], in which the trained classifier for a single time point is applied to every time point in a second set of data.

To compute statistical probability for all within-type, cross-decoding and time generalisation analyses, we used the Monte Carlo Cluster Statistics function in the CoSMoMVPA toolbox [41,42,43]. These statistics yielded a corrected p-value that represents the chance that the decoding accuracy could have come from a null distribution formed from 10,000 iterations [44]. These *p*-values were thresholded at *p*_corrected_ < 0.05 for significance.

## 3. Results

In this experiment, participants viewed rapid streams of images (Pattern Estimator) and series of imagery trials. In imagery trials, participants were presented with a sequence of four images (Vision) and then were cued to imagine one of the images (Imagery). We trained and tested multivariate classifiers to decode exemplar and category of the object in all three conditions, as well as tested the generalisation performance of classifiers between vision and imagery trials.

### 3.1. Behavioural Results

#### 3.1.1. Vividness of Visual Imagery Questionnaire

The VVIQ was scored out of 160, a sum of responses to each of the 16 questions on a five-point scale. The VVIQ was given to participants both with eyes open and closed [30]. The average overall score was 113 (*SD* = 15.93, range 82–150), similar to previously reported means [23,45,46]. Responses with eyes open (*M* = 56.44, *SD* = 8.54) were very similar to eyes closed (*M* = 57.69, *SD* = 10.28). The distribution of overall scores is shown in Supplementary Figure S2.

#### 3.1.2. Target Identification

**Figure 2:**
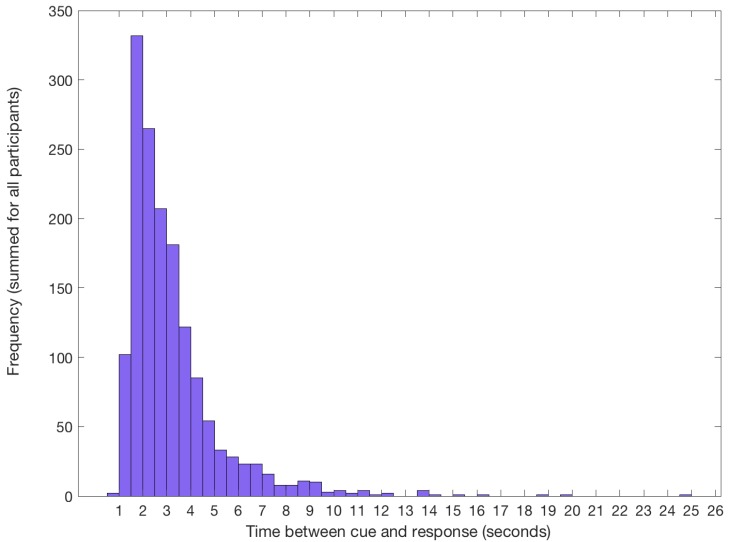
Frequency of response times from cue to imagery across all participants. Response time is taken from the onset of the numerical cue indicating the location of the target in the stream, until the participant voluntarily clicked the mouse. During this period, participants identified the correct target and began to imagine it on the screen.

To verify if participants were able to identify the target for imagery trials correctly, we examined their behavioural responses after each imagery sequence. Participants were able to accurately identify the target, with an average overall accuracy of 92% (*SD* = 4.40). Of the trials which were errors, most participants chose one of the four original images (67% of errors). Approximately a third of incorrect responses were to the flipped version of the target. This suggests participants successfully learned the basic characteristics of the target images and were not simply relying on a mnemonic strategy to complete the task. The mean response time from cue to imagery was 3.21 s (*SD* = 1.86), and the most frequent response time was between 1.5 and 2 s (Figure 2).

### 3.2. EEG Results

#### Significant Decoding of Image Category and Exemplars for Seen Images on Imagery Trials

**Figure 3:**
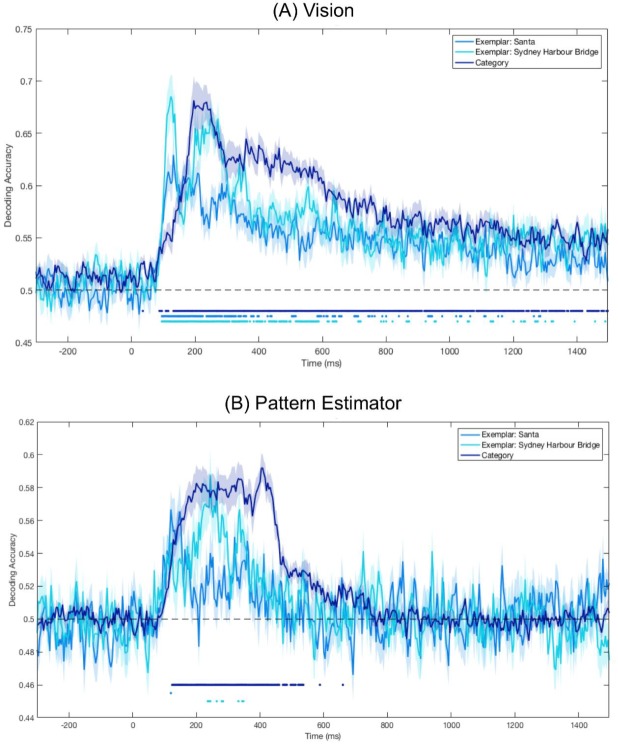
Mean decoding accuracy for Vision (**A**) and Pattern Estimator (**B**) images. Dots below plots indicate time points at which decoding was significantly above chance (*p* < 0.05). Shaded areas represent the standard error of the mean across subjects. (**A**) Vision: Decoding category and exemplar identity from the four target images presented in the experimental trials. (**B**) Pattern Estimator: Decoding category and exemplar identity from the 56 images presented in the fast streams at the beginning of each block; category decoding was based on all images in the stream classified by either face or place, and exemplar decoding was based only on the targets and modified targets.

To test whether category information was represented in visually displayed images, we trained and tested a classifier on the images seen during experimental trials (Vision). Category decoding was continuously above chance (*p*s < 0.05) after 88 ms (Figure 3), indicating patterns of brain activity for Santas and Sydney Harbour Bridges were distinguishable from this point. This above-chance decoding was sustained for the entire time the image was displayed. Continuous above-chance decoding began for both Santas and Sydney Harbour Bridges at 96 ms. Peak accuracy occurred at 132 ms for Santas, 124 ms for Sydney Harbour Bridges and at 196 ms for category decoding.

### 3.3. Significant Category Decoding in Pattern Estimator

To create a category classification model for imagery, we looked at patterns of brain activity while participants viewed images in the fast stream at the start of each block (Pattern Estimator). All images were labelled according to super-ordinate categories of ‘face’ or ‘place’. To assess the model’s utility, we cross-validated it on the Pattern Estimator trials. There was sustained above-chance category decoding from 124 ms after stimulus onset until approximately 535 ms after stimulus onset (Figure 3). The classifier was also able to distinguish between the two Sydney Harbour Bridge targets at several discrete time points between 236 ms and 348 ms after stimulus onset. There was no continuous above-chance decoding for Santas. Category decoding peaked at 404 ms after stimulus onset, at 244 ms for Sydney Harbour Bridges and at 120 ms for Santas.

### 3.4. No Significant Decoding for Imagery

**Figure 4:**
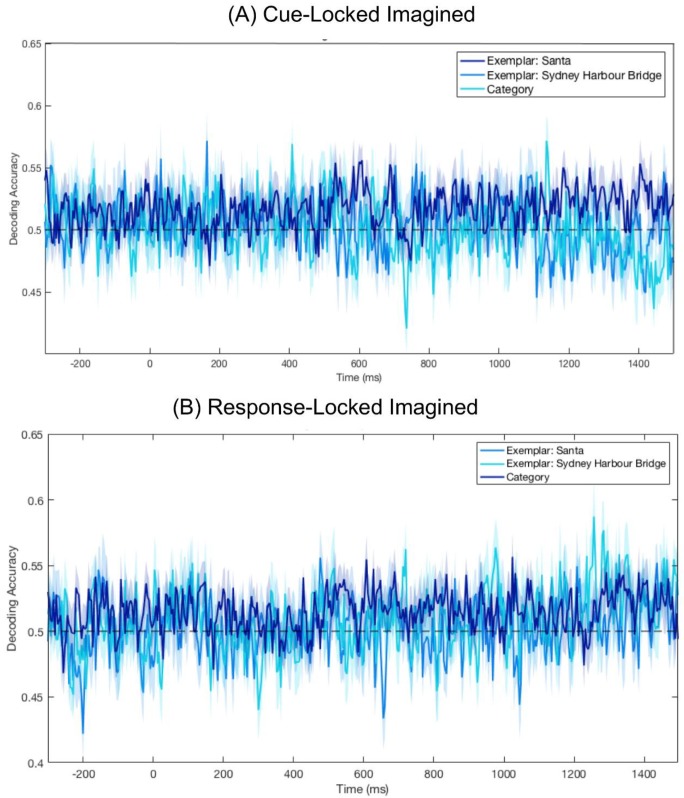
Mean decoding accuracy for Cue-Locked and Response-Locked Imagined epochs. The absence of dots below the plots indicates there were no points at which decoding was significantly above chance (*p*s > 0.05). Shaded areas represent the standard error of the mean across subjects. (**A**) Decoding accuracy centred on presentation of the numerical cue indicating the location of the target in the preceding stream. (**B**) Decoding accuracy centred on when participants clicked to advance to the imagining period.

To determine if category or exemplar information was decodable from imagined data, we trained and tested a classifier on the Cue- and Response-Locked Imagined epochs (Figure 4). Brain areas activated during imagery are known to vary between individuals [25], so we looked at imagery decoding on an individual subject basis. For each subject, we ran a permutation test in which the decoding procedure was run 1000 times, with category labels randomly assigned to the epochs. A *p*-value was calculated for each time point, based on the number of permutations with a greater decoding accuracy than the correct label decoding. We used the False Discovery Rate to correct for multiple comparisons. This test was conducted on both Response- and Cue-Locked epochs, and we found decoding was not significantly above chance for any individual at any time point for either Cue- or Response-Locked data (*p*s > 0.05).

To test whether there was any representational overlap in imagery and vision, we ran a cross-decoding analysis. We ran all pairwise combinations of vision and imagery; a classifier was trained to distinguish Santas from Harbour Bridges in the viewed stimuli (Pattern Estimator or Vision epochs) and was tested on imagery periods (Cue-Locked or Response-Locked). There were no significant periods of overlap for any cross-decoding involving imagined trials (*p*s > 0.05).

It could be that the processes in vision and imagery engage overlapping representations but at different times. To test this, we conducted a time generalisation analysis [40]. A classifier was trained on visual data (Pattern Estimator or Vision epochs) at each time point and then tested on imagined data (Cue- and Response-Locked) at every possible time point. There was no time point where decoding was significantly above chance for any combination of training and testing (all *p*s > 0.05), indicating there was no point where the patterns of brain activity during perceptually processed stimuli were present during imagery.

### 3.5. Differences in Vividness Did Not Affect Decoding Accuracy

Another possibility is that people with greater capacity for imagery have more decodable imagery representations. To investigate the effects of subjective imagery vividness on decoding accuracy, we grouped the participants as ‘high’ or ‘low’ imagery vividness based on a median split of their ‘eyes-open’ scores in the VVIQ. Two participants had the median score and were excluded from further analysis. We used the eyes-open score because it was the most relevant for the task at hand and makes our results comparable to prior MEG research [3], where only the eyes-open section was used. To see if there were any significant differences between the groups in any of the previously described analyses, we conducted a random-effects Monte Carlo statistic with 10,000 iterations to find where differences between the groups were significantly greater than zero. There was only one isolated point of significant differences between the two conditions, at 1484 ms, when the classifier was trained on Pattern Estimator data and tested on Response-Locked Imagery.

## 4. Discussion

The current study used time-series decoding to capture the precise temporal fluctuations underlying mental imagery. Based on prior MEG evidence showing the category and identity of imagined objects can be decoded, we expected successful category and exemplar decoding from imagery. However, contrary to our predictions, we were unable to detect any systematic representations of category or exemplar information during imagery. Based on previous evidence that imagery recruits similar neural networks to vision [4], we also anticipated overlapping patterns of neural activity when participants were viewing and imagining the same image. Although we were able to decode stimulus category and identity from visually processed stimuli, there were no time points where neural representations of vision and imagery were overlapping. Finally, we considered whether individual subject results might vary based on imagery vividness and found no systematic differences between subjects reporting high and low vividness. Overall, our findings suggest stimulus- and design-related factors may influence the chances of successfully decoding mental imagery.

To compare the overlap between imagery and visual processing, we first defined the temporal dynamics of visual processing for the images in this experiment. For stimuli presented as part of the imagery sequence (Vision), image category was predictable from approximately 100 ms after stimulus presentation until offset 1400 ms later. Exemplar decoding was also significant from 100 ms, albeit for less continuous time than category decoding, reflecting well-established evidence that both categories and exemplars evoke distinct patterns of brain activity [47]. For the Pattern Estimator, category decoding was significantly higher than chance from 100 ms until approximately 500 ms after stimulus onset. This extended period of decoding after stimulus offset supports recent evidence that multiple representations can co-exist in the brain [48,49].

In both visual conditions, exemplar decoding peaked earlier than category decoding. This reflects well-established evidence of increasing abstraction along the ventral visual pathway [47,50]. It also appears that decoding accuracy for Sydney Harbour Bridges is higher than for Santas, for both visual conditions (Vision and Pattern Estimator), though this pattern is less defined for the Pattern Estimator stimuli because of the low numbers of training and testing stimuli (4 of each exemplar per stream).

When the classifier trained on the visual stimuli was tested on imagery, there were no time points where the signal was sufficiently similar to accurately predict image category or identity. To investigate the possibility that the processes were not temporally aligned, we conducted a temporal generalisation analysis. There were no regular patterns of activity at the group level, indicating there was no overlap in representations at any point in the imagery period. Based on evidence that areas of activation during imagery vary across people (e.g., [25]), we examined results on the individual level. Patterns of individual decoding accuracy varied dramatically between subjects. Neither category nor exemplar decoding was significant at any time point for any individual. At face value, these results seem inconsistent with prior findings by Dijkstra and colleagues [3]. These differences primarily point to the difficulties of studying visual mental imagery, and the specific methodological characteristics required to obtain significant imagery decoding.

Several factors may have impacted our capacity to decode imagined mental representations, including decreased power compared to previous experiments and other methodological concerns. For example, it is possible that some aspects of the experimental design, such as the mouse-click, and the working memory requirements of four images, decreased the similarities between vision and imagery. However, a recent experiment used similar design choices and found that animacy information was available before information about visual features for perception, but this was in the reverse order for retrieval from memory [51]. Participants in the study used a mouse-click in a similar fashion to those in our study, indicating that this aspect of the design was not responsible for our null effects. Furthermore, it seems unlikely that working memory demands influenced our ability to decode imagery, because Linde-Domingo et al. [51] had arguably higher demands on working memory (eight word-image associations per block). One factor that might have influenced our ability to decode information during imagery, however, is the density of channels; most prior experiments use a greater number of channels than the 64 we had (128 EEG channels in [51] and 256 MEG channels in Dijkstra et al. (2018) [3]). This increased number of channels may provide better signal to noise ratio and increase chances of finding an effect [52]. An additional consideration is that individual variability in image generation would reduce the sensitivity of population statistics. Moreover, the temporal variability in an individual’s capacity to generate a mental image would further reduce individual effect sizes, as differences over time blur the signal.

We designed our experiment to check if participants used non-imagery-based strategies, to rule this out as a reason for significant decoding. We tested if participants were using a non-imagery strategy by including a superordinate category distinction with two exemplars in each category. We obtained response data after every trial with flipped images as distractors to test whether participants were using an imagery-based strategy. If participants were using a purely semantic label-based strategy, we would expect a similar number of responses for flipped and target images. However, only 0.33% of all responses were the flipped version of the target. These response patterns clearly show participants in our experiment were aware of the visual elements of the images rather than solely the semantic label. Due to the fundamentally introspective nature of mental imagery, there is no way to determine if participants are genuinely completing the imagery portion of the task. However, these response patterns point strongly to the use of an imagery-based strategy. Future experiments with similar hierarchical structure and more subtly modified response options (e.g., deleting or rotating a single element of the image, or changing colours of elements of the target images) could help determine whether this is a plausible theoretical explanation for our results.

Generation of mental imagery requires activation of complex, distributed systems [4]. Higher stimulus complexity increases the number of details that need to be recalled from memory. It therefore seems likely that the neural processes involved in viewing a static image are more temporally consistent than generating an image from memory, which is unlikely to follow a millisecond-aligned time-locked process. This is particularly apparent for complex stimuli which require more details, stored in potentially disparate locations, to generate vivid imagery. This same temporal blurring between trials from temporally misaligned processes is present in other prior studies [3], as it is somewhat inherent to the temporal specificity that decoding of time-series data provides.

Most previous experiments using complex visual scenes as imagery targets use an extensive training period prior to the study, relying on long-term memories of targets for imagery [28]. Although our participants completed a training period prior to EEG recording, slightly longer than those in Dijkstra and colleagues’ MEG study, it is possible [3] that participants might have experienced more vivid imagery if they had more exposure to the experimental images. Intuitively, it seems easier to imagine a highly familiar object such as an apple rather than a scene of Sydney Harbour because there are fewer details required to create an accurate representation. Mental images that are less vivid or less detailed are likely to generate weaker neural activation [53] and are less likely to fully resemble the details that are processed during vision. If the patterns are less distinct, a classifier is less likely to be able to identify reliable patterns of brain activity on which to base categorisation. To determine the effects of memory on imagery vividness and reliability, future study could compare the current results to a similar paradigm where subjects have extensive training prior to recording (e.g., participants are extensively questioned about characteristics of the image, or have to draw the main aspects to show awareness of details in the image).

As highlighted in recent research [54], individual differences in imagery generate increased variation between individuals. Pearson and Keogh (2019) provide a compelling argument for individual differences in strategy causing differences in imagery results. For example, only the visual working memory of ‘good imagers’ was affected by a manipulation of brightness (to interfere with low-level visual representations), indicating that only some participants were using imagery as a memory strategy [55,56].

In the current experiment, differences in visual working memory capacity, personal decision-making boundary, and memory strategy may have increased variation between participants. For example, individuals who report stronger imagery ability tend to use an imagery-based strategy on visual working memory tasks [57]. Features of both working memory and long-term memory (e.g., meaningfulness, familiarity) affect ratings of imagery vividness [58]. Furthermore, differences in strategy may also cause variation between individuals. These factors might also influence variability within a participant; increasing exposure to the four images might change neural responses over the course of the experiment as the participant recognises and remembers more image details.

Another source of variation may be the possibility that we captured a slightly different stage of imagery across individuals, as it is likely each person based the timing of their mouse clicks on a different threshold criterion for the point at which they had begun to imagine. Different strategies for identifying the target may have directed the focus of imagery. When asked informally at the conclusion of the experiment, all participants could explicitly describe their strategy for identifying the target. Most participants assigned a label to each image and mentally repeated these to remember the image order. The majority of strategies relied on structural characteristics, for example, “fat, tall, under, above”. Several participants also reported a direction-based strategy, for example, “top, bottom, centre, side” or “straight, side, face, body”, indicating the orientation of the main object in the image. Though there is no reliable way to compare decoding accuracy based on strategy, different strategies may direct focus on different aspects of the complex images (e.g., thinking of ‘face’ might make facial features salient, compared to labelling the same image as ‘fat’, drawing focus to body shape). These differences in strategy present another potential source of variation between subjects.

It is clear that capability to decode visual mental imagery is influenced by several factors, including vividness, memory and stimulus complexity. These factors do not affect imagery in isolation; they are inherently related. Better memory for the details of an image is likely to increase vividness. The number of details remembered by an individual is influenced not only by their memory capacity but also by the complexity of the stimulus and the number of details necessary to generate a vivid image. All these factors create variation in the processes used to generate mental imagery across both people and time [3,59]. The potential for MVPA techniques to analyse data at the individual level provides insight into the variation across subjects and highlights the need for future studies to consider patterns of data at an individual level to maximise the chances of obtaining clear signals from imagery.

## 5. Conclusions

In this study, we investigated how neural representations of mental imagery change over time. Our results suggest successful category decoding in earlier studies may be a result of better signal to noise ratio from a variety of factors, including individual variation. Variety in response times, imagery strategy and ability, in addition to fewer recording sensors may have reduced our power to find systematic patterns of neural activity during imagery. Furthermore, the interactions between stimulus complexity, working memory and imagery vividness may have increased this variation. Our results raise many questions for further investigation and demonstrate both the challenges and advantages associated with time-series decoding for EEG in investigating the introspective processes underlying mental imagery.

## Supplementary Materials

The following are available online at https://www.mdpi.com/2411-5150/3/4/53/s1.
