# Supplementary material for: Decoding Images in the Mind’s Eye: The Temporal Dynamics of Visual Imagery
Source: Vision (Basel). 2019 Oct 21;3(4):53. doi: 10.3390/vision3040053 (PMC6969936; doi:10.3390/vision3040053)
Supplement: Supplementary file 1 [file vision-03-00053-s001.pdf]

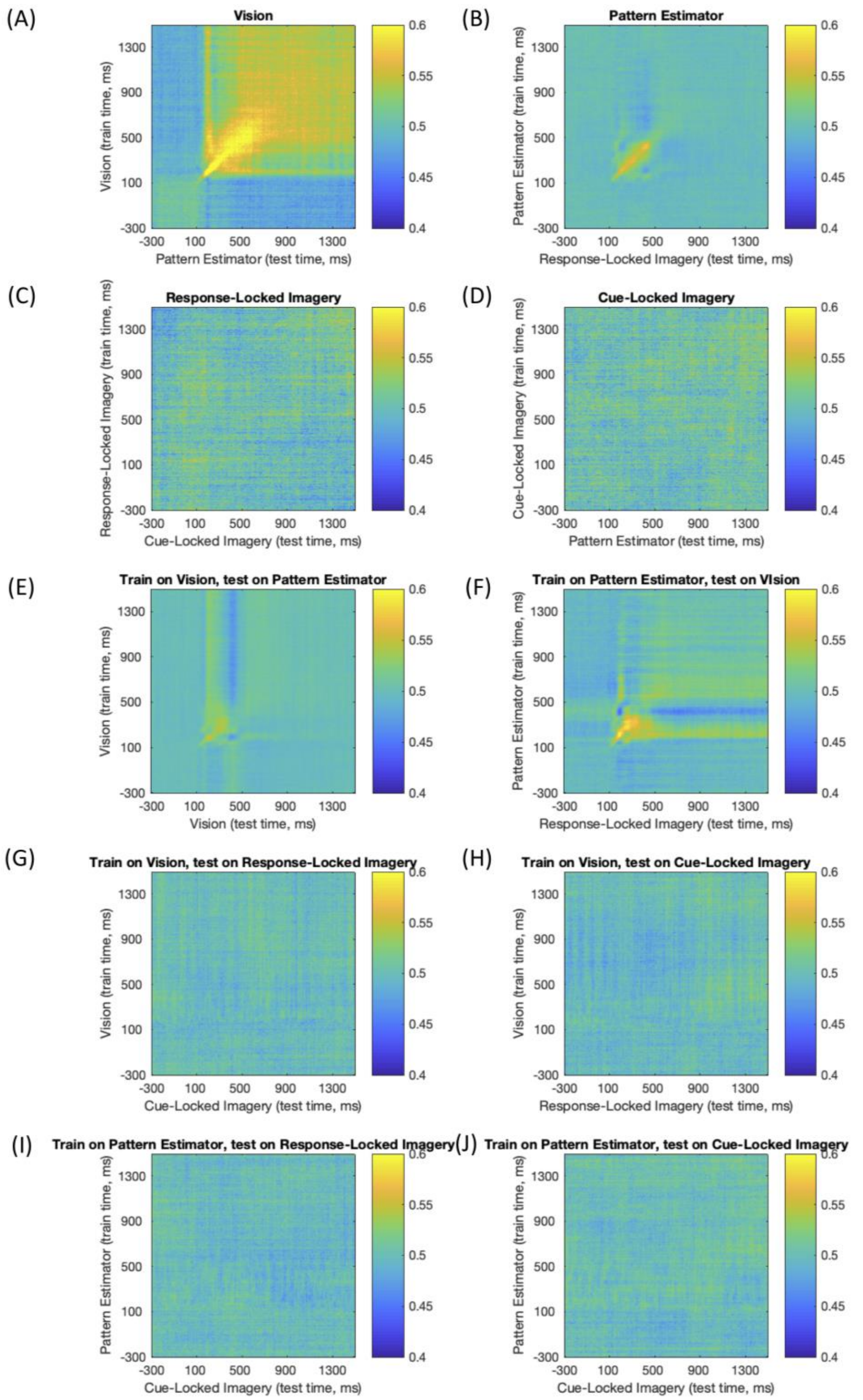


**Figure S1.** Temporal generalisation for cross-decoding analyses. For all plots, a classifier is trained on the time points on the y-axis, and then tested on all the points on the x-axis. The colour scale represents the decoding accuracy for each combination of time points. Each graph illustrates a different combination of training and testing data. For the within-epoch decoding (A-D), data were evenly split between training and testing sets. .

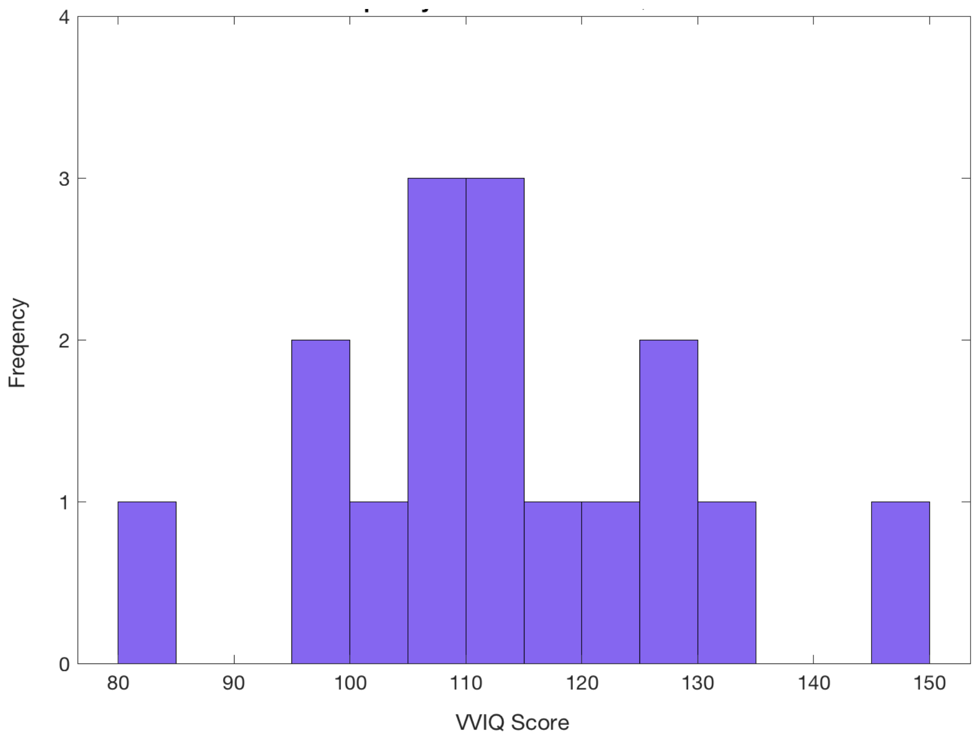


**Figure S2.** Frequency Distribution of scores in the Vividness of Visual Imagery Questionnaire overall scores. Scores are calculated out of a possible 160 by summing responses to each question completed with the eyes open and with the eyes closed. .
